# A randomized controlled trial on the effect of a silver carboxymethylcellulose dressing on surgical site infections after breast cancer surgery

**DOI:** 10.1371/journal.pone.0195715

**Published:** 2018-05-23

**Authors:** Gerson M. Struik, Wietske W. Vrijland, Erwin Birnie, Taco M. A. L. Klem

**Affiliations:** 1 Department of Surgery, Franciscus Gasthuis & Vlietland, Rotterdam, the Netherlands; 2 Department of Statistics and Education, Franciscus Gasthuis & Vlietland, Rotterdam, the Netherlands; 3 Division of Women and Baby, Department of Obstetrics and Gynecology, University Medical Center Utrecht, Utrecht University, Utrecht, the Netherlands; Department of Surgery and Cancer BioSurgery and Surgical Technology, St Mary’s Hospital, London

## Abstract

**Background:**

The incidence of surgical site infections (SSIs) after breast cancer surgery is relatively high; ranging from 3 to 19%. The role of wound dressings in the prevention of SSI after breast cancer surgery is unclear. This study compares a silver carboxymethylcellulose dressing (AQUACEL Ag Surgical (Aquacel) with standard wound dressing in SSI rate after breast cancer surgery.

**Patients and methods:**

A single-centre randomized controlled trial among women ≥18 years, diagnosed with breast cancer, undergoing breast conserving or ablative surgery, was conducted in a combined in and outpatient setting. The intervention was the use of Aquacel, compared with standard gauze dressing. Primary outcome measure was SSI following CDC criteria.

**Results:**

A total of 230 patients were analysed: 106 in the Aquacel group and 124 controls. Seven patients (6.6%) developed SSI in the Aquacel group and 16 patients (12.9%) in the control group (RR 0.51 [95% Confidence Interval (CI): 0.22–1.20]; p = 0.112; adjusted OR 0.49 [0.19–1.25] p = 0.135)). Unplanned exploratory subgroup analysis of breast conserving surgery patients showed that SSI rate was 1/56 (1.8%) in the Aquacel group vs. 7/65 (10.8%) in controls; adjusted OR 0.15 [0.02–1.31] p = 0.087. The Aquacel group showed better patient satisfaction (median 8 vs. 7 on a Numerical Rating Scale, p = 0.006), fewer dressing changes within 48 hours(adjusted OR 0.12 [0.05–0.27] p<0.001), fewer re-operations (0% vs. 3.2%, p = 0.062), and lower mean wound-related treatment costs, both in a high (€265.42 (SD = 908) vs. €470.65 (SD = 1223) [p<0.001]) and low (€59.12 (SD = 129) vs. €67.55 (SD = 172) [p<0.001]) attributable costs of SSI model.

**Conclusion:**

In this randomized controlled trial in women undergoing surgery for breast cancer, the use of AQUACEL Ag Surgical wound dressing did not significantly reduce the occurrence of SSIs compared to standard gauze dressing. The use of Aquacel resulted in significantly improved patient satisfaction, reduced dressing changes and reduced wound-related costs.

**Trial registration:**

www.trialregister.nl: NTR5840

## Introduction

### Background

Breast cancer is the most common malignancy among women in western countries, and one out of eight women will develop it during their lifetime. In the Netherlands, nearly 15,000 new cases are identified each year [1]. Breast cancer is the second cause of cancer-related deaths among women and the leading cause of disability-adjusted life years (DALYs) globally [2]. The majority of patients with breast cancer are treated surgically, amongst other treatment modalities. Although surgery of the breast is regarded as a clean procedure [3], a high incidence of surgical site infections (SSIs) is found, making it the most common complication [4]. Previous studies on SSIs in women after breast cancer surgery showed incidences ranging between 3% and 19% [4–7]. This is much higher than the expected 3.4% infection rate associated with clean surgical techniques [8].

SSIs are associated with considerable morbidity and reduced quality of life for patients. SSIs lead to extended hospital stays, re-admissions and re-operations, poor cosmetic results, delay in commencing adjuvant treatment, and they consequently result in additional costs and poorer outcomes [9–11]. Therefore, prevention of SSI has recently gained attention. A recent meta-analysis [12]identified several significant risk factors for SSI after breast cancer surgery, but the type of wound dressings was not evaluated in the included studies. A recent Cochrane Review [13]on the role of wound dressing in the prevention of SSIs revealed that in the current literature, there is no evidence that covering surgical wounds healing by primary intention with wound dressings reduces the rate of SSI, nor that any particular wound dressing is superior to another in this regard. Studies included in this review were mainly outdated and of poor quality. The authors concluded that decision-making should be based on dressing cost and the ability to deal with specific symptoms. High-quality research on the role of wound dressings in the prevention of SSIs is needed. As a result, the CDC guideline has no specific recommendation on the type of dressing or wearing time, except that primarily closed wounds should be sterile dressed for at least 24 to 48 hours [13, 14].

All sorts of wound dressings are available presently, containing different materials and using different application techniques. Characteristics of an ideal wound dressing are the ability to absorb and contain exudate without leakage, a lack of particulate contaminants left in the wound, thermal insulation, impermeability to water and bacteria, suitability with different skin closures (sutures, staples), avoidance of wound trauma on removal, little need for dressing change, provision of pain relief, cosmesis, comfort, and a positive effect on scar tissue formation [15,16].

AQUACEL Ag Surgical (Aquacel) is a type of wound dressing that is thought to meet these characteristics more than others: in-vitro tests showed that the silver in the dressing inhibits aerobic, anaerobic, gram-negative, and gram-positive bacteria, as well as yeast and fungi within 30 minutes [17,18]. The antibacterial activity lasts for 14 days [18]and it is occlusive. Several studies found that less changing of the dressing is needed. Furthermore, patient satisfaction was higher when a wound was treated with Aquacel [19, 20]. Despite Aquacel’s favourable characteristics, a randomized comparative study of Aquacel with other wound dressings after breast cancer surgery has not yet been performed.

### Objectives

Our aim in this study was to compare Aquacel with standard gauze wound dressing in the occurrence of SSI among women after breast cancer surgery. We hypothesise that Aquacel will reduce the occurrence of SSI in this particular group of patients.

## Patients and methods

### Trial design

This study was a prospective, open label, randomized, single center active controlled clinical study with a two arm 1:1 parallel group design. It was designed to assess the effectiveness of Aquacel in reducing the risk of SSIs in women after breast cancer surgery. The trial was set to establish the superiority of Aquacel to standard wound care. It was performed in a large secondary teaching hospital (Franciscus Gasthuis, Rotterdam), in a combined inpatient and outpatient setting. The inclusion period was between June 2013 and May 2016, with the last patient completing the 90-day follow-up in August 2016. The study protocol (dossier nr. NL42892.101.12) was reviewed and approved by the Medical Ethics Committee: Toetsingscommissie Wetenschappelijk Onderzoek Rotterdam (TWOR). After approval of the protocol the following changes in the protocol were made: Extension of the randomisation list to have the option to extend patient inclusion; addition of two exclusion criteria because of the anticipated differences of a priori SSI risk in neo-adjuvant chemotherapy and immediate reconstructive surgery; planning of an exploratory subgroup analysis of breast conserving surgery and mastectomy, for being clinically relevant.

The study was conducted according to the principles of the Declaration of Helsinki (version 10, October 2013) and in accordance with the Medical Research Involving Human Subjects Act (WMO).

### Participants

All women of age 18 years or above who were diagnosed with breast cancer, needing uni- or bilateral ablative or breast conserving surgery in our hospital, were considered eligible for inclusion in our study. Patients were only included after giving a written informed consent.

Patients were excluded if they had local inflammation or ulceration of the breast, previous breast surgery in the previous 3 months, use of antibiotics in the previous 2 weeks, neo-adjuvant chemotherapy, intended immediate reconstructive surgery, a known allergy for Aquacel or silver, and the inability to read or understand the Dutch language to give informed consent or fill out questionnaires.

### Interventions (surgical procedures, wound management and follow-up)

Included patients underwent ablative or conservative breast surgery, with or without an axillary procedure. All procedures were performed or supervised by senior surgeons with a case load of more than 50 per year. All patients received a single dose of intravenous antibiotics as recommended [21](cefazolin 1 gram, by hospital protocol) 1 hour before surgery. In accordance with the CDC guidelines, we considered bilateral procedures as two separate observations [22]. Drain management was performed according to the surgeon’s preference.

After surgery, wounds were cleaned with normal saline and patients received their allocated wound care: standard wound dressing, consisting of an eight layer woven cotton gauze fixed with adhesive tape, or Aquacel. AQUACEL Ag Surgical is a hydrocolloid dressing with hydrofiber technology that delivers ionic silver when it comes in contact with the wound (e.g., exudate). The occlusive dressing protects the skin surrounding the wound, by moisture retention. The material is soft and pliable and can therefore be adjusted to the size of the wound [17, 18].

Both standard wound dressing and Aquacel were kept in place for 7 days by protocol, unless saturated by excessive exudate. Between the 7^th^ and 10^th^ days after surgery, follow-up was scheduled at the outpatient clinic. Unblinded, the wound(s) were inspected on signs of an infection following CDC criteria [14, 22] by the independent surgeon or attending physician and any clinical diagnosis of SSI was made. Patients, who were unblinded, filled out a questionnaire on patient satisfaction. Re-admissions/operations, the occurrence of SSI diagnosis after the clinical assessment, and the use of antibiotics were scored 30 days after surgery in two ways: a blinded review of patients record by an independent physician and a telephone consult with the patient by an independent blinded nurse. Patient records were also checked for deep infections on the 90th postoperative day, by an independent blinded physician, to fulfil reporting guidelines [22].

### Outcomes

The primary outcome of this study was the incidence (risk) of SSI. Secondary outcome measures were patient satisfaction, the re-admission and re-operation rate, antibiotic use within 30 days, the need for changing the dressing within the first 48 hours, wearing time of first applied dressing, and costs. For managerial purposes, we added a cost analysis.

Aquacel costs €23.25 per dressing. Standard wound care costs €1.28 per dressing. Mean wound-related treatment costs were calculated with the following equation:

dressingprice*(1+proportiondressingchange<48h)+proportionSSI*attributablecostofSSI,

with attributable cost of SSI after breast surgery in a low (€510/$574[9]) and high(€3634/$4091[10]) cost model, based on the existing literature.

#### Assessment of SSI

**Fig 1:**
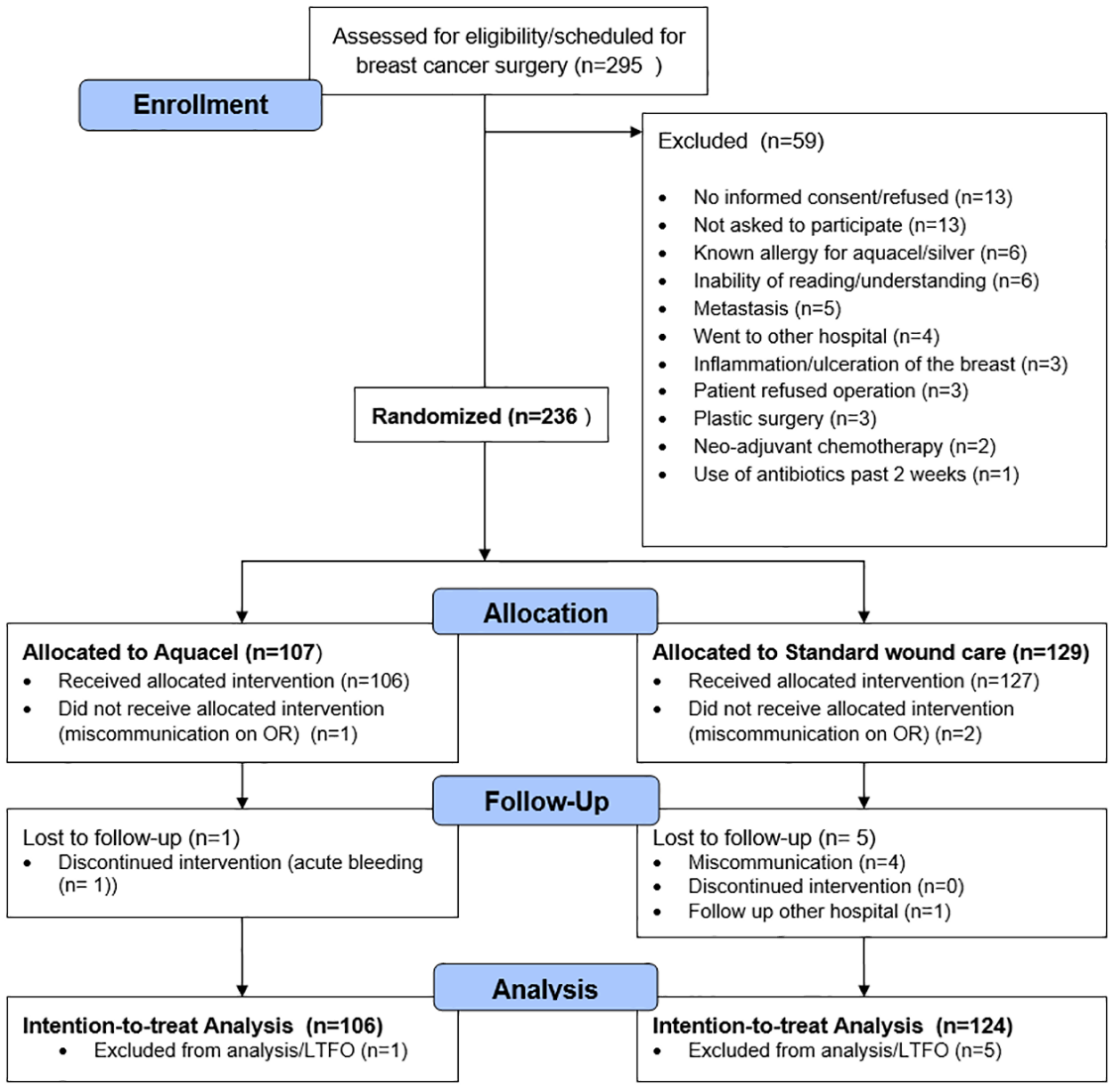
CDC criteria for an SSI [14,22]. ^a^Diagnosis of ‘cellulitis’ alone does not meet criterion 4 since 2010, but this change underestimates the infection rate and is not recommended to be used by Degnim [5].

SSI outcome was scored by a blinded and independent physician, using the CDC criteria following the reporting instructions after breast procedures: 30 days follow-up for superficial incisional SSI, and 90 days for a deep incisional SSI [14, 22] (Fig 1). Final scoring was based on the information captured from 1) the unblinded clinical observation recorded during the follow-up visit at day 7–10 after surgery, 2) the blinded review of patients record and telephone consult on day 30 after surgery, and 3) the blinded review of patients record after completing 90 day follow-up.

#### Patient satisfaction

Overall satisfaction regarding the wound dressing was scored by the patient on a 10-point numerical rating scale (NRS) from 0 (complete dissatisfaction) to 10 (complete satisfaction), on days 7–10 after the procedure. The 10-point NRS is commonly used in similar studies [23–28] and was externally validated to other patient surveys in the study by Keurentjes [29] who found correlations of 0.52 and 0.64, which can be interpreted as moderate to substantial correlation according to the Landis and Koch guidelines [30].

### Sample size

A 10% (12.5% to 2.5%) absolute reduction of occurrence of wound infection was considered to be clinically relevant. To reject the null hypothesis (risk of SSI is equal between the wound dressing strategies) with an accepted type 1 error of 5% (two-sided) and type 2 error of 20%, at least 106 patients per treatment arm would be required (randomization ratio 1:1) (www.sealedenveloppe.com, Chi^2^-test). No interim analysis was planned.

### Randomization (sequence generation, allocation, implementation) and blinding

A randomization list for up to 400 patients with an allocation ratio of 1:1 was computer generated (www.sealedenveloppe.com) with stratification by age>60 years of age, smoking, diabetes, use of corticosteroids, and the type of operation (lumpectomy vs mastectomy). The independent nurse created 400 instead of 212 numbers, under supervision of the study supervisor to compensate for and anticipate on the possibility to extend patient inclusion, any unplanned exploratory subgroup analysis and to guarantee a sufficient number of study numbers in each stratum. Patients were enrolled by physicians and group assignment was performed by the independent nurse. Allocation was performed on the day of surgery; for concealed allocation, the operation department was informed by the independent nurse just before applying the dressing. Surgeons were blinded for treatment allocation during surgery until the moment of applying the dressing, not during follow-up. Patients could not be blinded because of the nature of the intervention.

### Statistical methods

Statistical analysis was performed according to the intention-to-treat principle. Because protocol compliance was high, per-protocol analysis was avoided. As bilateral cases only occurred in two patients, adjusted statistics through repeated measurements analysis were not performed. Efforts were made to minimise missing data, by recalling patients not attending follow-up. If complete follow-up data were missing, patients were excluded from the analysis. Otherwise, patients were analysed only on available data. Differences in the baseline characteristics and the primary and secondary outcome measures between the allocated study groups were compared with the chi-squared test for nominal/ordinal variables (e.g. proportions of SSI, re-operations, early dressing change), the independent Student’s t-test for continuous variables with normal distributions (age), and the nonparametric Mann-Whitney U test for continuous variables with skewed distributions (e.g. operation duration, patient satisfaction, mean costs). Differences in outcome measures between groups were estimated using a logistic regression analysis (enter analysis) with the respective outcome measure as dependent variable and randomization factors and type of wound dressing as independent variables. Adjusted odds ratios with 95%CIs and p-values are reported. Differences in wearing time of the first dressing between groups were estimated using Cox regression analysis with dressing change as event, randomization factors as covariables and wearing time as time to event. Associations between the potential risk factors and the presence of SSI were quantified in terms of odds ratios (ORs with 95%CIs) and tested using binary multiple logistic regression analysis. Risk factors with a p-value <0.1 in univariate analysis were included in the logistic regression model (enter analysis). An unplanned exploratory subgroup analysis, as well as an unplanned effect modifier analysis was performed to detect differences in the primary outcome measure between breast conserving and ablative surgery, because of its high clinical relevance. Effect modification was modelled as an interaction effect of mode of breast surgery (lumpectomy vs. mastectomy) x allocated wound dressing (Aquacel vs. standard). A p-value < 0.05 (two-sided) was considered to be significant. Statistical analyses were performed using IBM-SPSS version 24 (IBM Corporation, Armonk, New York, USA).

## Results

### Participant flow

**Fig 2:**
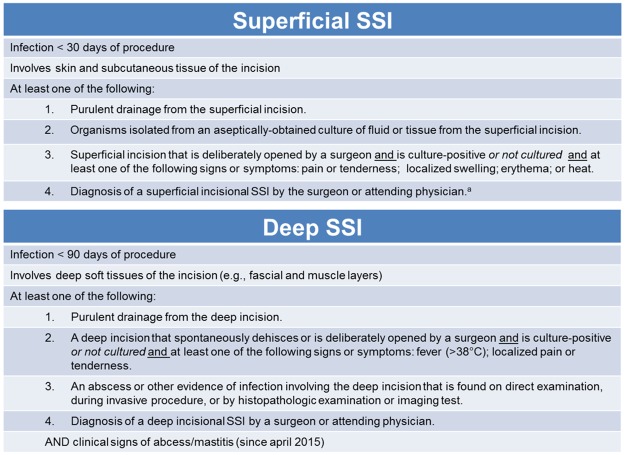
Patient flowchart (CONSORT).

A total of 295 patients underwent breast cancer surgery in our hospital, of which 59 patients were excluded. Fig 2 shows the study profile: 236 patients were randomized, 107 patients to the Aquacel group and 129 patients to the control group. Total loss to follow-up was 6/236 (2.5%), these patients were excluded from the analysis. Based on the available data in the medical records of these patients, no SSI occurred in these patients. Protocol compliance was 227/230 (98.7%). Finally, 230 patients were analysed on a intention-to-treat basis. There were no missing data in the primary outcome and secondary outcome measures (except dressing change), from which we conclude that analysis of available data only is not likely to have caused bias in the results.

Table 1 shows the baseline characteristics of patients. As expected, there were no differences between the groups.

**Table 1:** Baseline characteristics.

	Aquacel (n = 106)	Control group (n = 124)
Mean age, years(SD)	59 (12)	60 (13)
BMI >30	26/106 (24.5%)	29/124 (23.4%)
Diabetes	5/106 (4.7%)	6/124 (4.8%)
Current smoker	15/106 (14.2%)	19/124 (15.3%)
Corticosteroid use	1/106 (0.9%)	2/124 (1.6%)
ASA classification		
1	46/106 (43.4%)	47/124 (37.9%)
2	50/106 (47.2%)	68/124 (54.8%)
3	10/106 (9.4%)	9/124 (7.3%)
Positive *S*.*aureus* nasal culture	11/65 (16.9%)	16/83 (19.3%)
Type of surgery		
Lumpectomy + SLNB	52/106 (49.1%)	61/124 (49.2%)
Mastectomy +SLNB	35/106 (33.0%)	39/124 (31.5%)
Mastectomy+ALND	14/106 (13.2%)	20/124 (16.1%)
Lumpectomy + ALND	2/106 (1.9%)	3/124 (2.4%)
Lumpectomy	2/106 (1.9%)	1/124 (0.8%)
Mastectomy	1/106 (0.9%)	
Operation time, median in min (range)	78 (25–224)	73 (35–293)
Wounddrain	49/106 (46.2%)	54/124 (43.5%)
Drainage time in days, median (range)	2 (1–21)	2 (1–13)
Clinical stage (TNM)		
I	58/106 (54.7%)	65/124 (52.4%)
II	34/106 (32.1%)	45/124 (36.3%)
II	14/106 (13.2%)	14/124 (11.3%)

SLNB, sentinel lymph node biopsy; ALND, axillary lymph node dissection,

### Outcomes and estimation

Table 2 displays the outcome measures between the groups. A total of 7 patients (6.6%) developed an SSI in the Aquacel group, and 16 patients (12.9%) developed an SSI in the control group (RR 0.51 [95% Confidence Interval (CI): 0.22–1.20]; adjusted Odds Ratio (OR) 0.49 [0.19–1.25] p = 0.135). The majority of SSIs were superficial in both groups. In the Aquacel group, only one patient (0.9%) had a deep SSI, compared to four patients (3.2%) in the control group (RR 0.29 [CI: 0.03–2.58], adjusted OR 0.28 [0.03–2.54] p = 0.257).

**Table 2:** Outcome measures and comparison between the groups.

	Aquacel (n = 106)	Control (n = 124)	RR [CI]	Adjusted OR [CI]	p-value^a^
**SSI**					
Total	**7/106 (6.6%)**	**16/124 (12.9%)**	**0.51 [0.22–1.20]**	**0.49 [0.19–1.25]**	**0.135**
Superficial	6/106 (5.7%)	12/124 (9.7%)	0.59 [0.23–1.51]	0.58 [0.21–1.64]	0.306
Deep	1/106 (0.9%)	4/124 (3.2%)	0.29 [0.03–2.58]	0.28 [0.03–2.54]	0.257
**Patient Satisfaction**	**8 (1–10)**	**7 (0–10)**	**n.a**.	**n.a**.	**0.006**^b^
Re-admissions	7/106 (6.6%)	4/124 (3.2%)	2.05 [0.62–6.80]	2.21 [0.61–7.93]	0.225
**Re-operations**	**0/106 (0%)**	**4/124 (3.2%)**	**n.a**.	**n.a**.	**0.062**
Antibiotics use	12/106 (11.3%)	14/124 (11.3%)	1.00 [0.49–2.07]	1.04 [0.45–2.39]	0.934
**Dressing change<48h**	**9/94 (9.6%)**	**45/98 (45.9%)**	**0.21 [0.11–0.40]**	**0.12 [0.05–0.27]**	**<0.001**
**Wearing time first dressing**	**7 (1–7)**	**3 (0–7)**	**n.a**.	**0.42 [0.31–0.57]**	**<0.001**^c^

^a^*p*-values were calculated using the logistic regression model, unless stated otherwise,

^b^ Mann Whitney U test,

^c^Cox regression model

Furthermore, the Aquacel group scored significantly better than controls on patient satisfaction (median score of 8 vs. 7 [p = 0.006] and need for changing the dressing within the first 48 hours (9.6%, vs. 45.9% [RR 0.21 (CI: 0.11–0.40),adjusted OR 0.12 [0.05–0.27] p<0.001). For the outcome measure dressing change, a sensitivity analysis was performed showing an observed difference between the allocated groups of 9/94 (9.6%) and 45/98 (45.9%); a minimal estimated difference of 21/106 (19.8%) Aquacel vs 45/124 (36.3%) control; and a maximal estimated difference of 9/106 (8.5%) vs 71/124 (57.3%).

For the whole group lowering the need for early changing of the dressing was associated with higher patient satisfaction (median score of 8 in the ‘no early change’ vs. 7 in the ‘early change’ group; p = 0.003. A non-significant reduction in re-operation rate was found (0% vs. 3.2%, p = 0.062). Of the 23 patients with an SSI in this study, 15 had a positive bacterial culture result. Among these patients, *Staphylococcus aureus* and *Pseudomonas aeruginosa* were found most frequently, in 10/15 cases (67%) and in 3/15 cases (20%), respectively. Other culture results can be found in Table 3.

**Table 3:** Microbiological culture results of SSI cases.

Micro-organism	Overall (n = 15) ^a^	Aquacel (n = 6) ^a^	Control (n = 9) ^a^
***Staphylococcus aureus***	**10/15 (66.7%)**	**4/6 (66.7%)**	**6/9 (66.7%)**
***Pseudomonas aeruginosa***	**3/15 (20.0%)**	1/6 (16.7%)	**2/9 (22.2%)**
*Serratia marcescens*	2/15 (13.3%)	1/6 (16.7%)	1/9 (11.1%)
*β-hemolytic Streptococcus group B*	1/15 (6.7%)		1/9 (11.1%)
*β-hemolytic Streptococcus group A*	1/15 (6.7%)	1/6 (16.7%)	
*Haemophilus parainfluenzae*	1/15 (6.7%)		1/9 (11.1%)
*Citrobacter freundii*	1/15 (6.7%)	1/6 (16.7%)	
*Enterobacter aerogenes*	1/15 (6.7%)		1/9 (11.1%)
*Acinetobacter baumannii*	1/15 (6.7%)		1/9 (11.1%)

^a^% do not add to 100%. One SSI patient may have two or more microorganisms as the causative agent.

### Ancillary analyses

#### Logistic regression analysis

The following potential risk factors for SSI were analysed: Age > 60 years, BMI>30, presence of diabetes mellitus, current smoking, corticosteroid use, positive *s*.*aureus* nasal culture, ASA class (2 or more), use of post-operative drain, prolonged drainage time (≥3 days), operation time, histological diagnosis, high clinical TNM stage (3 or more), dressing change within the first 48 hours, type of surgery (lump yes/no), any axillary procedure yes/no (no was only 3 patients) and ALND vs no ALND Univariate analysis identified the following potential risk factors for SSI (p>0.1): use of post-operative drain (p = 0.016), prolonged drainage time (≥3 days) (p = 0.002), operation time (p = 0.050), high clinical TNM stage (3 or more) (p = 0.015), dressing change within the first 48 hours (p = 0.036), any axillary procedure (p = 0.013)Of these risk factors, prolonged drainage time (≥3 days) (adjusted OR 5.722 [1.406–23.297] p = 0.015)) and dressing change within the first 48 hours (adjusted OR 2.979 [1.022–8.685] p = 0.046) were found to be independent risk factors for SSIs in a logistic regression analysis.

#### Subgroup and effect modifier analysis

Unplanned exploratory subgroup analysis of type of surgery was performed. In the subgroup of breast conserving surgery, the SSI rate was 1/56 (1.8%) in the Aquacel group vs. 7/65 (10.8%) in controls, RR 0.17 [CI: 0.03–0.99], adjusted OR 0.15 [0.02–1.31] p = 0.087. This would result in a number needed to treat (NNT) of 11.1 [CI 5.8–145.4] patients to prevent one SSI in this subgroup. Patient satisfaction was significantly higher in the Aquacel group (median score 8 vs. 7, p = 0.003) and the need for changing the dressing within the first 48 hours was lower (6.1% vs. 37.3%, p<0.001). The large reduction of SSI risk was not found in the group of patients undergoing mastectomy: 6/50 (12%) vs. 9/59 (15.3%); RR 0.89 [CI: 0.56–1.40], adjusted OR 0.77 [0.25–2.35] p = 0.647.

Effect modifier analysis showed that the interaction breast conserving surgery*Aquacel resulted in an adjusted OR 0.13 [0.02–1.14] p = 0.065, suggesting a trend that the use of Aquacel wound dressing is more effective in reducing SSIs when applied to patients who received breast conserving therapy.

#### Costs

Mean wound-related treatment costs were significantly lower in the Aquacel group than in the controls, both in the high (€265.42 (SD = 908) vs. €470.65 (SD = 1223) [p<0.001]) and low (€59.12 (SD = 129) vs. €67.55 (SD = 172) [p<0.001]) attributable costs of the SSI model.

### Harms

There were no important harms or unintended effects in both groups.

## Discussion

We found an SSI risk of 6.6% for the Aquacel group and 12.9% for the control group. The SSI rate in the control group is comparable to previous studies in the recent literature using CDC criteria for definition of SSI [5–7]. The incidence of SSI after breast cancer surgery is high, and although breast surgery is regarded as a clean surgical procedure, SSI is a relatively common complication. In our study we found that the use of AQUACEL AG Surgical dressing approximately reduces 50% of the incidence of SSI compared with standard dressing in women after surgery for breast cancer (RR 0.51), although we were not able to detect a significant difference. Exploring the effect in the subgroup of patients undergoing breast conserving surgery showed a relative reduction of 83% (RR 0.17) that was also not significant. Furthermore, with Aquacel, the need to change dressings within the first 48 hours was significantly lower, as was the need for re-operation (though not as significantly). Patient satisfaction was higher and the mean costs were lower with Aquacel, both significantly. Overall, Aquacel improves patient satisfaction and reduces dressing changes, but did not significantly reduce SSI risk in this particular patient group.

### Strengths and limitations

To our knowledge, this is the first well-designed RCT that investigates the effect of a silver-containing dressing on SSI rates after breast cancer surgery. Major strengths of this study, apart from its randomized design, are the inclusion of all types of breast cancer surgery, the use of very strict criteria for SSI (CDC) as the primary outcome, complete (90 days) follow-up, very few patients being lost to follow-up, and high protocol compliance.

One study weakness is the fact that during follow-up, patients and surgeons or attending physicians were not blinded when assessing satisfaction and infectious signs. This could potentially lead to optimistic satisfaction scoring by the patient for the new therapy, resulting in an incorrect significant difference. The lack of blinding by the physicians during follow-up could potentially lead to underreporting the clinical diagnosis of infection in the interventional group. However, scoring of the outcome measure SSI itself was done by a blinded physician, not involved in the surgical procedures or follow-up clinical observations. Furthermore, we aimed to minimize the risk of bias in outcome assessment by the physician and nurse by using very strict criteria for both clinical observation scoring and SSI outcome scoring (CDC). A second weakness is that although the SSI rate in the control group was estimated quite accurate, detecting a 10% absolute reduction starting from 12.5% was rather ambitious. The definition of a minimal clinically relevant reduction was extensively debated in our study group at the time of protocol development. Eventually we opted for a 10% absolute reduction. It is a rather conservative estimate in the sense that every surgeon will support that a 10% absolute reduction is clinically meaningful. The disadvantage of this conservative approach is that the study is likely to overlook smaller, but maybe also clinically relevant, risk reductions. Our study showed a relative risk reduction of approximately 50%. A total of 694 patients would have been needed to demonstrate a significant reduction of this size. A third limitation is that the large randomisation list resulted in a slight skewness of treatment allocation. However, comparison of baseline characteristics showed that treatment groups were comparable and the randomisation was not subverted. Lastly, SSI can result in a delay in adjuvant treatment and consequently an impaired oncological outcome[23, 31]. However, we did not specifically analyse the impact of this delay.

### Interpretation and generalisability

Generalisation of our findings should be done with caution, as we acknowledge the fact that there is a lack of clear evidence about the value of dressings in surgical practice, and some surgeons use glue or no dressings[32], as opposed to the simple dressing which we have used as control intervention.

Interpretation of SSI reduction rates should be balanced against the nature of the intervention, the setting and the related morbidity, quality of life and costs. There is no guideline providing any strict recommendation on how this interpretation can be achieved. In recent years it has been increasingly recommended by several authors [33–36] to also judge the clinical relevance of study findings. In our study, there is no disadvantage/harm for patients through the intervention and there is proven benefit in terms of patient satisfaction and costs. Therefore, given the fact that our study is underpowered for the detection of a minimal clinically meaningful difference, i.e. 5%, we consider the rather large effect size of SSI reduction in our study to be relevant.

The discrepancy in treatment effect found by the exploratory subgroup analysis between breast conserving surgery and ablative surgery might be explained by the fact that other factors than the type of wound dressing contribute more to the development of SSI in ablative surgery: compromised vascularisation of skin and subcutaneous tissue by extensive dissection, seroma and hematoma formation, and prolonged drainage time [4, 12, 37]. It seems that with the importance of these factors, the type of wound dressing plays a negligible or modest role in reducing the risk of infection after breast ablative surgery. Research in that patient group should focus on reducing these specific risk factors.

As expected, using Aquacel lowered the need for changing the dressing. Early change of the dressing (within the first 48 hours after surgery) was shown to be an independent risk factor for SSI occurrence in this study. This could be an explanation of the lower SSI rate in the Aquacel group, besides the antibacterial effect of the silver. Recommendation of the CDC to sterile dress primarily closed wounds for at least 24–48 hours could be extended to not change the dressing in the first 48 hours, as early change of the dressing was shown to be an independent risk factor for SSI in our study. Furthermore, lowering the need for early changing of the dressing was associated with an improvement in patient satisfaction of more than 5%, which in literature on quality of life and utility is considered to be a relevant difference [38, 39].

Our findings are highly relevant for healthcare providers, with significant differences in favour of the Aquacel group on two of the recently proposed outcome measures to assess wound management by Elliot[32]: patient satisfaction with the dressing and dressing removal. Based on our exploratory subgroup analysis, the treatment effect of silver-containing dressings on SSI rates might be different between breast conserving surgery and mastectomy. Our results stimulate early drain removal and discourage the early change of dressings. Furthermore, reduced costs and improved patient satisfaction are very relevant in healthcare nowadays. Finally, our study confirms the findings of studies in orthopaedic surgery [20, 40], that found that Aquacel improved patient satisfaction and reduced dressing changes, but could not confirm a significant reduction of SSI.

In summary, clinicians should be aware of the difference in risk factors for SSI between breast conserving and ablative surgery and the role of Aquacel dressings in improving patient satisfaction, reducing dressing changes and possibly reducing SSI after breast cancer surgery.

## Conclusion

In this randomized controlled trial in women undergoing surgery for breast cancer, the use of AQUACEL Ag Surgical wound dressing did not significantly reduce the occurrence of SSIs compared to standard gauze dressing. The use of Aquacel resulted in significantly improved patient satisfaction, reduced dressing changes and reduced wound-related costs.

## Supporting information

S1 File Outpatient clinic clinical observation scoring form—English version. (PDF)

S2 File Outpatient clinic clinical observation scoring form—Dutch version. (PDF)

S3 File Study protocol. (PDF)

S4 File Consort 2010 checklist. (PDF)

## References

[1] Netherlands Cancer Registry 2015 [updated 2015; cited 2016 2016/6/12]. http://www.cijfersoverkanker.nl/p=557b111870359.

[2] Collaboration Global Burden of Disease Cancer. The Global Burden of Cancer 2013. JAMA Oncol. 2015;1(4):505–27. doi: 10.1001/jamaoncol.2015.0735 26181261 10.1001/jamaoncol.2015.0735 PMC4500822

[3] Haley RW, Culver DH, Morgan WM, White JW, Emori TG, Hooton TM. Identifying patients at high risk of surgical wound infection. A simple multivariate index of patient susceptibility and wound contamination. Am J Epidemiol.1985:206–15. 4014116 10.1093/oxfordjournals.aje.a113991

[4] El-Tamer MB, Ward BM, Schifftner T, Neumayer L, Khuri S, Henderson W. Morbidity and mortality following breast cancer surgery in women: national benchmarks for standards of care. Ann Surg. 2007:665–71. 10.1097/01.sla.0000245833.48399.9a PMC1877061 17457156

[5] Degnim AC, Throckmorton AD, Boostrom SY, Boughey JC, Holifield A, Baddour LM, et al Surgical site infection after breast surgery: impact of 2010 CDC reporting guidelines. Ann Surg Oncol. 2012;19(13):4099–103. doi: 10.1245/s10434-012-2448-6 22732837 10.1245/s10434-012-2448-6 PMC3926500

[6] Gulluoglu BM, Guler SA, Ugurlu MU, Culha G. Efficacy of prophylactic antibiotic administration for breast cancer surgery in overweight or obese patients: a randomized controlled trial. Ann Surg. 2013 1;257(1):37–43. doi: 10.1097/SLA.0b013e31826d832d 23001082 10.1097/SLA.0b013e31826d832d

[7] Williams N, Sweetland H, Goyal S, Ivins N, Leaper DJ. Randomized trial of antimicrobial-coated sutures to prevent surgical site infection after breast cancer surgery. Surg Infect (Larchmt). 2011 12;12(6):469–74. 22142317 10.1089/sur.2011.045

[8] Vazquez-Aragon P, Lizan-Garcia M, Cascales-Sanchez P, Villar-Canovas MT, Garcia-Olmo D. Nosocomial infection and related risk factors in a general surgery service: a prospective study. J Infect. 2003:17–22. 12504604 10.1053/jinf.2002.1073

[9] Reilly J, Twaddle S, McIntosh J, Kean L. An economic analysis of surgical wound infection. J Hosp Infect. 2001:245–9. 10.1053/jhin.2001.1086 11740871

[10] Olsen MA, Chu-Ongsakul S, Brandt KE, Dietz JR, Mayfield J, Fraser VJ. Hospital-associated costs due to surgical site infection after breast surgery. Arch Surg. 2008;143(1):53–60; discussion 61. doi: 10.1001/archsurg.2007.11 18209153 10.1001/archsurg.2007.11

[11] Avritscher EB, Cooksley CD, Rolston KV, Swint JM, Delclos GL, Franzini L, et al Serious postoperative infections following resection of common solid tumors: outcomes, costs, and impact of hospital surgical volume. Support Care Cancer. 2014;22(2):527–35. doi: 10.1007/s00520-013-2006-1 24141699 10.1007/s00520-013-2006-1

[12] Xue DQ, Qian C, Yang L, Wang XF. Risk factors for surgical site infections after breast surgery: a systematic review and meta-analysis. Eur J Surg Oncol. 2012;38(5):375–81. doi: 10.1016/j.ejso.2012.02.179 22421530 10.1016/j.ejso.2012.02.179

[13] Dumville JC, Gray TA, Walter CJ, Sharp CA, Page T. Dressings for the prevention of surgical site infection. Cochrane Database Syst Rev. 2014. 10.1002/14651858.CD003091.pub3 25178020

[14] Mangram AJ, Horan TC, Pearson ML, Silver LC, Jarvis WR. Guideline for Prevention of Surgical Site Infection, 1999. Centers for Disease Control and Prevention (CDC) Hospital Infection Control Practices Advisory Committee. Am J Infect Control. 1999:97–132. 10196487

[15] BNF. British National Formulary. Appendix 8: Wound management products and elastic hosiery 2010.

[16] NICE. NICE clinical guideline 74. Surgical site infection: Prevention and treatment of surgical site infection [Internet]. 2008. https://www.nice.org.uk/guidance/cg74.

[17] Bowler PG. Microbicidal properties of a silver-containing hydrofiber dressing against a variety of burn wound pathogens. J Burn Care Rehabil. 2004:192–6. 15091147 10.1097/01.bcr.0000112331.72232.1b

[18] Barnea Y, Weiss J, Gur E. A review of the applications of the hydrofiber dressing with silver (Aquacel Ag(^®^)) in wound care. Ther Clin Risk Manag. 2010;6:21–7. 20169033 PMC2817785

[19] Clarke JV, Deakin AH, Dillon JM, Emmerson S, Kinninmonth AWG. A prospective clinical audit of a new dressing design for lower limb arthroplasty wounds. J Wound Care. 2009;18(1):5–11. doi: 10.12968/jowc.2009.18.1.32128 19131911 10.12968/jowc.2009.18.1.32128

[20] Springer BD, Beaver WB, Griffin WL, Mason JB, Odum SM. Role of Surgical Dressings in Total Joint Arthroplasty: A Randomized Controlled Trial. Am J Orthop 2015;44(9):415–20. 26372751

[21] Jones DJ, Bunn F, Bell-Syer SV. Prophylactic antibiotics to prevent surgical site infection after breast cancer surgery. Cochrane Database Syst Rev. 2014(3):CD005360 doi: 10.1002/14651858.CD005360.pub4 24609957 10.1002/14651858.CD005360.pub4

[22] Prevention Centers for Disease Control and. Surgical Site Infection (SSI) Event. Guidelines and procedures for monitoring SSI. [updated 2016; cited 2016 31–05]. http://www.cdc.gov/nhsn/PDFs/pscManual/9pscSSIcurrent.pdf.

[23] Cannavo M, Fairbrother G, Owen D, Ingle J, Lumley T. A comparison of dressings in the management of surgical abdominal wounds. J Wound Care, 1998, 7(2), 57–62. 9543974 10.12968/jowc.1998.7.2.57

[24] van Berckel MM, Bosma NH, Hageman MG, Ring D, Vranceanu AM, The Correlation Between a Numerical Rating Scale of Patient Satisfaction With Current Management of an Upper Extremity Disorder and a General Measure of Satisfaction With the Medical Visit. Hand (N Y), 2017 12(2): p. 202–206. 28344535 10.1177/1558944716662019 PMC5349416

[25] Viciano V, Castera JE, Medrano J, Aguiló J, Torro J, Botella MG, et al Effect of hydrocolloid dressings on healing by second intention after excision of pilonidal sinus. Eur J Surg. 2000 3;166(3):229–32. 10755338 10.1080/110241500750009339

[26] Berry DP, Bale S, Harding KG. Dressings for treating cavity wounds. J Wound Care. 1996 1;5(1):10–7. 8697123 10.12968/jowc.1996.5.1.10

[27] Langlois J, Zaoui A, Ozil C, Courpied JP, Anract P, Hamadouche M. Randomized controlled trial of conventional versus modern surgical dressings following primary total hip and knee replacement. Int Orthop. 2015 7;39(7):1315–9. doi: 10.1007/s00264-015-2726-6 25787680 10.1007/s00264-015-2726-6

[28] Specht K, Kjaersgaard-Andersen P, Kehlet H, Wedderkopp N, Pedersen BD, High patient satisfaction in 445 patients who underwent fast-track hip or knee replacement. Acta Orthop, 2015 86(6): p. 702–7 doi: 10.3109/17453674.2015.1063910 26109124 10.3109/17453674.2015.1063910 PMC4750770

[29] Keurentjes JC, Patient acceptable symptom states after total hip or knee replacement at mid-term follow-up: Thresholds of the Oxford hip and knee scores. 2014 3(1): p. 7–13. 10.1302/2046-3758.31.2000141 PMC3928564 24421318

[30] Landis JR, Koch GG, The measurement of observer agreement for categorical data. Biometrics, 1977 33(1): p. 159–74.31. 843571

[31] Tsoutsou PG, Belkacemi Y, Gligorov J, Kuten A, Boussen H, Bese N, et al Optimal sequence of implied modalities in the adjuvant setting of breast cancer treatment: an update on issues to consider. Oncologist. 2010;15(11):1169–78. doi: 10.1634/theoncologist.2010-0187 21041378 10.1634/theoncologist.2010-0187 PMC3227907

[32] Elliott D, Developing outcome measures assessing wound management and patient experience: a mixed methods study. BMJ Open. 2017;7(11):e016155 doi: 10.1136/bmjopen-2017-016155 29180591 10.1136/bmjopen-2017-016155 PMC5719294

[33] Grocott HP. Understanding the Significance of Aerosolized Vasodilator Use in Pulmonary Hypertension: What Is Numerically, Statistically, and Clinically Meaningful? Anesth Analg 2017; 125(6): 2167. 10.1213/ANE.0000000000002519 28953496

[34] Hung M, Bounsanga J, Voss MW. Interpretation of correlations in clinical research. Postgrad Med 2017; 129(8): 902–906. doi: 10.1080/00325481.2017.1383820 28936887 10.1080/00325481.2017.1383820 PMC6130913

[35] Mellis C. Lies, damned lies and statistics: Clinical importance versus statistical significance in research. Paediatr Respir Rev 2018; 25: 88–93. doi: 10.1016/j.prrv.2017.02.002 28341168 10.1016/j.prrv.2017.02.002

[36] Allan GM, Finley CR, McCormack J, Kumar V, Kwong S, Braschi E et al Are potentially clinically meaningful benefits misinterpreted in cardiovascular randomized trials? A systematic examination of statistical significance, clinical significance, and authors’ conclusions. BMC Med 2017; 15(1): 58 doi: 10.1186/s12916-017-0821-9 28316281 10.1186/s12916-017-0821-9 PMC5357813

[37] Mukesh MB, Barnett G, Cumming J, Wilkinson JS, Moody AM, Wilson C, et al Association of breast tumour bed seroma with post-operative complications and late normal tissue toxicity: results from the Cambridge Breast IMRT trial. Eur J Surg Oncol. 2012;38(10):918–24. doi: 10.1016/j.ejso.2012.05.008 22704052 10.1016/j.ejso.2012.05.008

[38] Walters SJ, Brazier JE, Comparison of the minimally important difference for two health state utility measures: EQ-5D and SF-6D. Quality of Life Research 2005;14:1523–32. 16110932 10.1007/s11136-004-7713-0

[39] Horsman J, Furlong W, Feeny D, Torrance G, The Health Utilities Index (HUI): concepts, measurement properties and applications Health Quality of LifeOutcomes, 2003;1:5440. 10.1186/1477-7525-1-54 PMC293474 14613568

[40] Cai J, Karam JA, Parvizi J, Smith EB, Sharkey PF. Aquacel surgical dressing reduces the rate of acute PJI following total joint arthroplasty: a case-control study. J Arthroplasty. 2014;29(6):1098–100. doi: 10.1016/j.arth.2013.11.012 24405622 10.1016/j.arth.2013.11.012

