# Supplementary material for: A randomized controlled trial on the effect of a silver carboxymethylcellulose dressing on surgical site infections after breast cancer surgery
Source: PLoS One. 2018 May 23;13(5):e0195715. doi: 10.1371/journal.pone.0195715 (PMC5965831; doi:10.1371/journal.pone.0195715)
Supplement: S1 File — (PDF) [file pone.0195715.s001.pdf]

[ ] Study ID<br>AQUACEL AG Surgical Study<br>*Outpatient visit (7-10 days post-op) – scoring form*	Patient identification sticker

Date: ___________________ Physician: _________________________

Specialized nurse : _________________________

Dressing change : YES / NO (circle); if yes, after how many days: ___________

<u>CDC-criteria</u>		YES	NO
1. Purulent drainage from incision		☐	☐
2. Cultured		☐	☐
Positive result (to fill out later)		☐	☐
3. Symptoms	Pain or tenderness	☐	☐
	Lokalized swelling	☐	☐
	Erythema	☐	☐
	Heat	☐	☐
	Fever (>38°C)	☐	☐
4. Deliberately opening of wound by surgeon		☐	☐
5. Spontaneous wound dehiscence		☐	☐
6. Clinical signs of abcess/mastitis		☐	☐

Diagnosis of SSI by surgeon or attending physician (circle): JA / NEE

In case of SSI (suspicion): superficial / deep (circle)

Start antibiotics (circle): YES / NO

If yes, specify: _________________; duration ______days

<u>Patient satisfaction</u>: please rate the wound dressing (circle)

0 1 2 3 4 5 6 7 8 9 10

complete dissatisfaction — complete satisfaction
