# Supplementary material for: A randomized controlled trial on the effect of a silver carboxymethylcellulose dressing on surgical site infections after breast cancer surgery
Source: PLoS One. 2018 May 23;13(5):e0195715. doi: 10.1371/journal.pone.0195715 (PMC5965831; doi:10.1371/journal.pone.0195715)
Supplement: S2 File — (PDF) [file pone.0195715.s002.pdf]

Studie ID

Patiëntensticker

AQUACEL AG Surgical Study

Polibezoek (7-10 dagen post-op) – score formulier

Datum: ____________________ Arts: ___________________________

mammacareverpleegkundige: ___________________________

Verbandwissel : JA / NEE (omcirkel); zo ja, na hoeveel dagen: ____________

CDC-criteria		JA	NEE
1. Pus uit incisie		☐	☐
2. Wondkweek afgenomen		☐	☐
Positief (later in te vullen)		☐	☐
3. Symptomen	Pijn of gevoeligheid	☐	☐
	Lokale zwelling	☐	☐
	Roodheid	☐	☐
	Warmte	☐	☐
	Koorts (>38)	☐	☐
4. Openen van wond		☐	☐
5. Spontane wonddehiscentie		☐	☐
6. Klinische tekenen van abces/mastitis		☐	☐

Klinische diagnose wondinfectie door chirurg (omcirkel): JA / NEE

In het geval van (verdenking) wondinfectie: oppervlakkig / diep (circle)

Start antibiotica (omcirkel): JA / NEE

Zo ja, welke: ___________________; duur ______dagen

Patiënttevredenheid: geef cijfer voor wondverband (omcirkel)

0 1 2 3 4 5 6 7 8 9 10

Volledig Ontevreden — Volledig Tevreden
