# Supplementary material for: A randomized controlled trial on the effect of a silver carboxymethylcellulose dressing on surgical site infections after breast cancer surgery
Source: PLoS One. 2018 May 23;13(5):e0195715. doi: 10.1371/journal.pone.0195715 (PMC5965831; doi:10.1371/journal.pone.0195715)
Supplement: S3 File — (PDF) [file pone.0195715.s003.pdf]

Title	**Aquacel Ag Surgical Study**
Version	2
Date	25 January 2012
Project leaders	T.M.A.L. Klem, MD, PhD, surgeon<br>W.W. Vrijland, MD, PhD, surgeon
Principal investigator	A.P. Jairam, MD, surgical resident
Sponsor	Convatec
Independent physician	U. Biter, MD, surgeon

Table of contents

Introduction	3
Objective	4
Study design and methods	5
Study population	7
Sample size determination, statistics and randomization	8
Aquacel Ag Surgical	9
Literature	10

Introduction

One out of eight women will develop breast cancer and most of these women need breast surgery. After breast cancer surgery the wound is covered by a ‘standard’ wound dressing; each clinic has its own standard wound therapy. These standard wound bandages can result in undesirable consequences, such as postoperative wound infection and blister formation due to, for example, changing the bandage on regular base. In this study, Aquacel Ag Surgical dressing will be compared with standard wound dressing in breast cancer patients undergoing breast ablative therapy or breast conserving therapy. The primary goal of this study is to determine if Aquacel Ag Surgical reduces postoperative wound infections.

Objective

This study will compare standard wound dressing to Aquacel Ag Surgical in patients undergoing BAT or BCT; in both treatment arms the patients will be randomized for one of the wound dressings. Primary outcome is postoperative wound infection.

Study design and Methods

This study will be a prospective, Randomized Controlled Trial, comparing standard wound dressing to Aquacel Ag Surgical. This is not a double-blinded study. The study will take place in the Sint Franciscus Gasthuis in Rotterdam and it will be performed in a (out-patient) clinical setting.

Patients diagnosed with breast cancer will visit the outpatient clinic. Here, they will receive an explanation about the study and a patient information letter. Patients will be included if they satisfy the inclusion criteria and sign the informed consent form. Patients will undergo breast surgery; breast ablative therapy or breast conservative therapy.
After surgery, patients will get one of the wound dressing therapies: standard wound dressing, consisting of a gauze fixated with adhesive tape, or Aquacel Ag Surgical. After the 10th day of surgery, follow up will take place at the outpatient clinic by an independent specialized nurse and the surgeon or the resident.

At the outpatient clinic the wound will be inspected according to the following classification.

Classification of post-operative wound infection:
I Superficial infections: infection of the skin or subcutaneous tissue which arises within 30 days after surgery and has one of the following findings; the presence of redness, pus, pain, tenderness and/or local oedema.
II Deep infections: infection of the muscle or fascia which arises within 30 days after surgery and has one of the following findings: pus, or the presence of an abscess, or an temperature of more than 38 degrees, or one of the following clinical characteristics: pain, tenderness, local oedema, redness.
III Infections of organs or anatomical spaces that are open or anatomical spaces that are manipulated during surgery. That means infection of that part of the anatomical space which arises within 30 days after surgery and has at least one of the following characteristics: pus coming out of the drain, or an abscess, or a positive culture infection.

On the first postoperative outpatient clinic visit, an independent specialized ‘breastcare’ nurse will collect data of each patient. There is no more follow up needed for this study. However,

wound complications will be reported if a patient will return within 30 days with a wound infection

An independent physician will be available for questions about the study. The study coordinator, A.P. Jairam, will supervise the inclusion of the patients and collecting and processing the results.

The proposed starting date is 1 March 2013.

Study population

Patients will be recruited from the outpatient clinic of the surgical department of the Sint Franciscus Gasthuis, Rotterdam, in the Netherlands. Women with breast cancer, who are undergoing surgical therapy; Breast Ablative Therapy (BAT) or Breast Conserving Therapy (BCT), are eligible patients for the trial.
This study requires 106 patients in each treatment arm (BAT and BCT). They will be randomized for standard wound dressing or Aquacel Ag Surgical dressing.
A period of 18 months should be sufficient to include the needed number of patients.

Inclusion criteria

- women diagnosed with breast cancer
- Age > 18 years

Exclusion criteria

- local inflammation or ulceration of the breast
- prior breast surgery past 3 months
- known allergy for Aquacel Ag Surgical
- the inability of reading/understanding not enabling to give informed consent or to fill out questionnaires
- use of antibiotics past 2 weeks

Sample size determination and statistics

For the power calculation, superiority of the primary (binary) endpoint, postoperative wound infection (POWI), in the treatment arm is assumed (H1 hypothesis). Previous surgical (pilot) studies and observations with Aquacel Ag Surgical showed (an often non significant) reduction of POWI between 5-10%. In our hospital we have 12-13% POWI after breast surgery. To detect a clinically relevant absolute difference of 10% in POWI (with a two-sided significance level of 0·05 and a power of 80 per cent, it is calculated that 106 patients per study arm should be required. Total sample size would be 212 patients. No interim analysis is planned. Data will be compared using the $\chi 2$ test. The primary endpoint is analysed on an intention-to-treat (ITT) basis. For confirmation, per-protocol (PP) analysis will also be carried out.

Two-sided $P < 0{\cdot}05$ is considered statistically significant. Statistical analyses will be performed using IBM-SPSS version 20 (IBM Corporation, Armonk, New York, USA)
Stratification is performed for the following criteria:

- Age (younger than 60 years versus 60 years and older)
- Axillary node dissection (yes / no)
- Diabetes (yes / no)
- Corticosteroids (yes / no)
- Operation (lumpectomy versus ablation)

Randomization

A randomization list for 212 patients with an allocation ratio of 1:1 has been computer generated with Sealed Enveloppe Company (www.sealedenveloppe.com) with stratification by age, axillary node dissection, diabetes, use of corticosteroids and type of operation. For concealed allocation an external randomization bureau is called the day of surgery after informed consent had been obtained. Patients are informed about the treatment assignment after surgery.

Aquacel Ag Surgical

Aquacel Ag Surgical dressing is developed for patients with wounds with a risk of wound infection. It delivers ionic silver when the wound (exudate) is in contact with the bandage. It also protects the skin surrounded by the wound. The material of Aquacel Ag Surgical is soft and can therefore be transformed and adjusted to the size of the wound.
In-vitro tests showed that the silver in the dressing kill bacteria within 30 minutes. The antibacterial activity will last for 7 days.
Several studies found out that, because of the antibacterial activity, less changing of the dressing is needed. Patients experience less pain and fear during the changing and they give the appearance of the wound treated with Aquacel Ag Surgical a better score.
As a result of less changing, the costs of this dressing are reduced compared to other dressings used in the studies.

The study is non invasive, not painful, and there is no extra burden for the patients; it is without risks. Therefore there is no need for insurance for the subjects.

Literature

1. Surgical site infections following colorectal cancer surgery: a randomized prospective trial comparing common and advanced antimicrobial dressing containing ionic silver.

   Biffi R, Fattori L, Bertani E, Radice D, Rotmensz N, Misitano P, Cenciarelli S, Chiappa A, Tadini L, Mancini M, Pesenti G, Andreoni B, Nespo

   World J Surg Oncol. 2012 May 23;10:94. doi: 10.1186/1477-7819-10-94.

   PMID: 22621779

2. A randomized comparison study of Aquacel Ag and Glucan II as donor site dressings with regard to healing time, cosmesis, infection rate, a patient's perceived pain: a pilot study.

   Bailey S, Carmean M, Cinat M, Burton K, Lane C, Malinoski D.

   J Burn Care Res. 2011 Nov-Dec;32(6):627-32. doi: 10.1097/BCR.0b013e31822dc409.

   PMID: 21844815

3. A review of the applications of the hydrofiber dressing with silver (Aquacel Ag) in wound care.

   Barnea Y, Weiss J, Gur E.

   Ther Clin Risk Manag. 2010 Feb 2;6:21-7.

   PMID: 20169033
